# Integrated single-cell and bulk RNA sequencing analysis reveals *ACACA* as a potential prognostic and immunotherapeutic biomarker across cancers

**DOI:** 10.3389/fimmu.2025.1599223

**Published:** 2025-10-15

**Authors:** Haihua He, Zhen Zhang, Leifeng Chen, Fushan Gao, Yuze Wu, Lina Yi, Fei Shao, Yibo Gao, Jie He

**Affiliations:** ^1^ Cancer Center, Renmin Hospital of Wuhan University, Wuhan, China; ^2^ Department of Thoracic Surgery, National Cancer Center/National Clinical Research Center for Cancer/Cancer Hospital, Chinese Academy of Medical Sciences and Peking Union Medical College, Beijing, China; ^3^ Laboratory of Translational Medicine, National Cancer Center/National Clinical Research Center for Cancer/Cancer Hospital, Chinese Academy of Medical Sciences and Peking Union Medical College, Beijing, China; ^4^ State Key Laboratory of Molecular Oncology, National Cancer Center/National Clinical Research Center for Cancer/Cancer Hospital, Chinese Academy of Medical Sciences and Peking Union Medical College, Beijing, China; ^5^ Department of Oncology, The Second Affiliated Hospital of Nanchang University, Nanchang, Jiangxi, China; ^6^ Precision Oncology Medicine Center, The Second Affiliated Hospital of Nanchang University, Nanchang, Jiangxi, China; ^7^ Medical Center for Cardiovascular Diseases, Neurological Diseases and Tumors of Jiangxi Province, The Second Affiliated Hospital of Nanchang University, Nanchang, China; ^8^ Department of Cancer Prevention and Control, National Cancer Center/National Clinical Research Center for Cancer/Cancer Hospital, Chinese Academy of Medical Sciences and Peking Union Medical College, Beijing, China; ^9^ Institute of Cancer Research, Henan Academy of Innovations in Medical Sciences, Zhengzhou, Henan, China; ^10^ Central Laboratory & Shenzhen Key Laboratory of Epigenetics and Precision Medicine for Cancers, National Clinical Research Center for Cancer/Cancer Hospital & Shenzhen Hospital, Chinese Academy of Medical Sciences and Peking Union Medical College, Shenzhen, China; ^11^ Department of Gastroenterology, Shanxi Province Cancer Hospital/Shanxi Hospital Affiliated to Cancers Hospital, Chinese Academy of Medical Sciences/Cancer Hospital Affiliated to Shanxi Medical University, Taiyuan, China

**Keywords:** Acetyl-CoA carboxylase alpha (*ACACA*), pan-cancer analysis, single-cell analysis, tumor microenvironment, drug resistance

## Abstract

**Background:**

Acetyl-CoA carboxylase alpha (*ACACA*), a crucial rate-limiting enzyme governing *de novo* biosynthesis of fatty acids, drives oncogenic metabolic reprogramming in diverse malignancies. However, the multiomics investigation and immunological implications of *ACACA* across cancers remain unclear.

**Methods:**

We performed a comprehensive pan-cancer analysis of *ACACA* via transcriptomic, proteomic, and clinical data from The Cancer Genome Atlas (TCGA), Clinical Proteomic Tumor Analysis Consortium (CPTAC), and the Human Protein Atlas (HPA) databases. Then, single-cell RNA sequencing acquired from the Gene Expression Omnibus (GEO) database was employed to map the expression pattern of *ACACA* in the tumor microenvironment (TME). Subsequently, functional validation experiments were conducted in lung cancer and sarcoma cells.

**Results:**

High *ACACA* expression was associated with poor survival in various cancers, particularly those exhibiting dysregulated lipid metabolism. Immune profiling revealed that elevated *ACACA* expression was associated with low infiltration of CD8^+^ T cells and activated natural killer (NK) cells. Single-cell analysis of lung adenocarcinoma revealed that *ACACA* was expressed predominantly within malignant cells and contributed to an immunosuppressive microenvironment through migration inhibitory factor (MIF) signaling and the extracellular matrix (ECM) remodeling pathway. Furthermore, *in vitro* studies demonstrated that ACACA inhibition suppresses fatty acid synthesis and tumor growth in lung cancer and sarcoma cells.

**Conclusions:**

Our study establishes *ACACA* as a key metabolic regulator that links lipid metabolism to immune evasion and drug resistance, highlighting its potential as a promising therapeutic target across cancers.

## 1 Introduction

Lipid metabolism is widely acknowledged as a core cellular process underpinning bioenergetic demands, membrane biogenesis, signal transduction, and regulation of the tumor microenvironment (TME) (1, 2). Characterized by high metabolic demands, tumor cells rely on enhanced fatty acid biosynthesis to fuel their rapid growth and maintain viability. Tumor cells exhibit characteristic changes in the expression levels and functional dynamics of enzymes critical for lipid metabolism, including acetyl-CoA carboxylase 1 (ACC1), ATP citrate lyase (ACLY) and fatty acid synthase (FASN) (3, 4). Lipid metabolic reprogramming and specific lipid signatures have emerged as potential biomarkers for disease assessment, prognosis prediction, and treatment response monitoring.

The enzyme encoded by the *ACACA* gene is Acetyl-CoA Carboxylase 1(ACC1), which facilitates the conversion of acetyl-CoA into malonyl-CoA through a carboxylation reaction, serving as the critical first-step enzymatic reaction in fatty acid biosynthesis (5). Structurally, ACC1 is a multifunctional enzyme with domains like biotin carboxylase (BC) and carboxyltransferase (CT), whose polymerization and dissociation affect the enzyme’s activity (6, 7). Cells exhibit adaptive regulation of fatty acid synthesis and oxidation, adjusting these processes according to different metabolic conditions, which underscores the complexity and importance of *ACACA* in maintaining cellular homeostasis and adaptability (8). Given *ACACA*’s significant involvement in the synthesis of fatty acids, it has emerged as a potential target for various metabolic disorders, including non-alcoholic hepatitis (NASH), obesity, and diabetes (9, 10).

In addition to its canonical involvement in lipid biosynthesis, increasing evidence in tumor and non-tumor contexts has shown that *ACACA* has pleiotropic functions in metabolism and immune regulation, modulating immune cell functionality, inflammatory responses, macrophage polarization, and overall immune surveillance (11–14). Among them, studies on *ACACA* in tumors have made remarkable progress. In prostate cancer, *ACACA* downregulation reduces ATP production, disrupts mitochondrial function, and increases ROS levels (15). In breast cancer, *ACACA* drives resistance to aromatase inhibitors in estrogen-deprived cells (16). Moreover, in murine models with liver-specific ACC knockout, carcinogen exposure doubles the incidence of tumor formation, collectively underscoring *ACACA*’s oncogenic capacity (17). These findings highlight the significance of further exploration of *ACACA* in tumors. The comprehensive multiomics profiling and immunological implications of *ACACA* across various cancer types have yet to be fully elucidated.

To fully assess the role of *ACACA* across cancers, we employed bioinformatic techniques to analyze *ACACA* expression data across several cancer databases, including The Cancer Genome Atlas (TCGA), Cancer Cell Line Encyclopedia (CCLE), and Clinical Proteomic Tumor Analysis Consortium (CPTAC). First, we conducted a comprehensive analysis to investigate the correlations of *ACACA* expression levels with key clinical outcomes, we also examined its involvement in immune cell infiltration and the tumor-immune landscape. Subsequently, pathway enrichment analysis was carried out to explore tits potential functions associated with *ACACA*. Then, single-cell analysis was leveraged to delineate *ACACA* expression patterns within both malignant and immune cell subgroups. Finally, functional experiments were conducted to confirm its role in lung cancer and sarcoma.

## 2 Materials and methods

### 2.1 Data acquisition

We obtained transcriptome profiles and sample data from TCGA (https://portal.gdc.cancer.gov/), CCLE (http://www.sites.broadinstitute.org/ccle) and the Genotype-Tissue Expression (GTEx; (https://gtexportal.org/home/). Protein characterizations were obtained from The Cancer Proteome Atlas (TPCA; http://bioinformatics.mdanderson.org/main/TCPA: Overview) and CPTAC (https://pdc.cancer.gov/pdc/browse). Additionally, UCSC Xena databases (https://xenabrowser.net/datapages/) also provided most of these datasets we used.

### 2.2 Differentially Expressed Genes analysis and prognostic analysis

CCLE RNA-seq data underwent TPM normalization and were filtered to retain genes expressed in >80% samples. Differential expression analyses were conducted via the limma package in R with empirical Bayes moderation. *ACACA* upregulation was defined by adj. p<0.001 and log_2_FC>1.5. Optimal survival-based cutoff values for *ACACA* mRNA expression were identified using the “surv_cutpoint” R function, patient were categorized into two distinct subgroups, namely *ACACA*-High and *ACACA*-Low, according to the established threshold values. The limma package facilitated the analysis of differential gene expression, with significance defined as adjusted P < 0.05. Univariate Cox proportional hazards regression (using survival R package, v3.7.0) was used to calculate hazard ratios (HRs) for associations between *ACACA* expression and four survival endpoints: overall survival (OS), disease specific survival (DSS), disease free interval (DFI) and progression free interval (PFI). The “survfit” function was employed to construct Kaplan-Meier survival curves, and survival differences between groups were statistically evaluated via log-rank tests. The receiver operating characteristic (ROC) curves were computed using the pROC package (v1.18.0) to assess the predictive performance of the models, the evaluation of diagnostic performance was conducted by calculating the area under the curve (AUC).

### 2.3 Immune infiltration analysis

To assess the Stromal and Immune Cells in Malignant Tumors (ESTIMATE) score, we utilized the “estimate” R package. Additionally, we explored associations between immune cell infiltration and gene expression patterns across various cancer types, we employed several computational deconvolution methods to analyze the correlation between these biological parameters (TIMER, xCell, MCP-counter, EPIC and CIBERSORT). Bubble plots generated through ggplot2 (v3.5.1) visualized associations between *ACACA* expression patterns and immune cell infiltration potential, with statistical significance defined by Benjamini-Hochberg adjusted p-values. TIDE (Tumor Immune Dysfunction and Exclusion) score is an algorithm for predicting responses to immunotherapy by analyzing tumor gene expression data, which calculating a comprehensive score by evaluating the two major mechanisms of tumor immune escape.

### 2.4 Single-cell RNA sequencing analysis

The scRNA-seq dataset was retrived from the Gene Expression Omnibus (GEO) under accession number GSE131907 (http://www.ncbi.nlm.nih.gov/geo). Tumor and matched normal samples were subjected to computational analysis via Seurat (v5.0) within R. The expression matrices were first normalized with the “NormalizeData” function, followed by identification of various features with the “FindVariableFeatures” function, and the “ScaleData” function was used for data scaling. Subsequently. Principal component analysis (PCA) and cell clustering were performed. Nonlinear manifold embedding was visualized through uniform manifold approximation and projection (UMAP) topology. Differential gene expression profiling across clusters was executed via the FindAllMarkers function employing a Wilcoxon rank-sum test framework. Cellular annotation leverages canonical lineage markers curated from peer-reviewed ontologies (Cell Marker database v2.0) and references carcinogenesis literature. Intercellular communication networks were deconvoluted using CellChat (v2.1.2) with the human ligand-receptor interaction repository (CellChatDB.human), which quantifies autocrine/paracrine signaling modalities including secreted factors, extracellular matrix interactions, and direct membrane contact pathways.

### 2.5 Drug sensitivity analysis

Information regarding drug sensitivity and gene expression was obtained from the Genomics of Drug Sensitivity in Cancer database (GDSC, https://www.cancerrxgene.org/). Drugs with an FDR < 0.05 were deemed statistically significant. Bubble plots were created via the R package ggplot2 to display associations between *ACACA* expression, drug half-maximal inhibitory concentrations (IC_50_), and their FDR values(v3.5.1).

### 2.6 Human samples and immunohistochemistry

We collected 41 paraffin-embedded sections of tumor tissues and paired normal tissues from the Cancer Hospital of the Chinese Academy of Medical Sciences. These patients were diagnosed with LUAD and underwent surgical resection during 2015 and 2016. The project obtained approval from the Ethics Committee of the Cancer Hospital of the Chinese Academy of Medical Sciences and acquired patients’ informed consent. These paraffin-embedded samples were stained with anti-ACC1 antibody (1:200 dilution; Cell Signaling Technology, #3676). Immunohistochemistry staining was performed as previously described. We evaluated tissue protein expression levels using the Histochemistry score (H-score), specifically calculated as: ​​H-score = Σ(*pi* × *i*)​, *i*​​ represents the staining intensity grade: 0 (Negative), 1 (Weak positive), 2 (Moderate positive), 3 (Strong positive), and *pi* denotes the percentage of positively stained cells within each intensity category.

### 2.7 Cell lines and siRNA transfection assay

The experimental design included four human lung adenocarcinoma cell models (PC9, HCC827, A549, and H1299), with Beas-2B bronchial epithelial cells used as non-malignant controls. The osimertinib-resistant cells PC9OR and HCC827OR were established by the method of increasing the drug concentration step by step. The IC_50_ values of Osimertinib in the parental cells versus resistant cells were quantified using the CCK-8 assay (**Supplementary Figure 5E**). Gene-specific siRNA duplexes (Shanghai GenePharma Co.) targeting ACACA were transfected via jetPRIME^®^ reagent following the manufacturer’s reverse transfection protocol. The siACACA siRNA sequences (5’-3’) were as follows: siRNA#1 (GCAGCUAUGUUCAGAGAAUTT), siRNA#2 (GCUCAUACACUUCUGAAUATT). Both preliminary and subsequent functional validation experiments demonstrated no significant difference in *ACACA* silencing efficiency between the two *ACACA* siRNAs.

### 2.8 Immunocytochemistry assay

Cellular samples were cultured via confocal imaging disher for 24 hours under standard growth conditions. After immobilization, permeabilization, and blocking, primary antibody incubation was performed with rabbit monoclonal anti-ACC1 (1:200 dilution; Cell Signaling Technology, #3676) and mouse anti-α-tubulin (1:200 dilution; Sigma-Aldrich, T6074) at 4°C for 16 hours. After washes with PBS-T (0.1% Tween-20), the samples were exposed to species-matched secondary antibodies conjugated to Alexa Fluor 488 (anti-rabbit) and Alexa Fluor 594 (anti-mouse) for 2 hours. Nuclear counterstaining employed DAPI for 5 min before mounting. Confocal imaging was performed, and images were captured.

### 2.9 RT-PCR and RT-qPCR

Total RNA was isolated from the cellular samples using a DNA/RNA extraction kit (RK30153, ABclonal Biotechnology Co., Ltd., Wuhan, China) following the manufacturer’s protocol. Next, the RNA was reverse transcribed into complementary DNA (cDNA) using the ABScript II cDNA First-Strand Synthesis Kit (Takara).Then, the 7500 real-time PCR system from Applied Biosystems was employed for qPCR analysis, utilizing the SYBR Premix Ex Taq kit manufactured by Takara. Chemically synthesized primers were obtained from Generay (Shanghai, China), and their sequences are presented below. The forward primer for *ACACA* (5’->3’): is AGGAGCTGTCTATTCGGGGT, and the reverse primer (5’->3’) is GGTCGCTCAGCCTGTACTTT. The *ACTB* forward primer(5’->3’) is CTCGCCTTTGCCGATCC, and the reverse primer (5’->3’) is ATCCTTCTGACCCATGCCC.

### 2.10 CCK8 assay

Using the CCK-8 kit (RM02823, ABclonal Biotechnology Co., Ltd., Wuhan, China). In accordance with the experimental protocol, the cells were digested, resuspended, counted before and then plated into 96-well plates. At predefined time points (0, 24, 48, 72, and 96 hours), each well received 10 μl of CCK-8 solution. Following 2-hour incubation, absorbance at 450 nm was measured using a microplate reader. The average absorbance value of 5 wells was calculated, and each assay was replicated three times. Sterile PBS blanks and cell-free medium controls were included for background correction.

### 2.11 Transwell assay

Metastatic potential was quantified through modified Boyden chamber assays using Corning BioCoat chambers (8-μm pore, #3422). In the migration experiments, a total of 5×10^3^ cells were plated into the upper compartment containning serum-free medium, while 600 μL of complete medium was added to the lower compartment. Prior to cell seeding in the invasion assay, 50 μL of diluted BD Matrigel matrix was applied to the upper chamber membrane. After the appropriate incubation period, the cells were incubated with 4% paraformaldehyde, and the fixed cells underwent coloration with 0.1% crystal violet solution. Migration patterns were observed, and image were acquired through microscopic examination. The Cells in five random fields per chamber were counted.

### 2.12 Western blot

A protease inhibitor cocktail was added into the RIPA buffer. Following lysis, total protein concentration was quantified using the Pierce BCA kit (Thermo Scientific, #23227). Proteins were separated by 8% SDS-PAGE. All other steps are carried out in accordance with the standard instructions. The primary antibodies included rabbit monoclonal anti-ACC1 (Cell Signaling Technology, #3676), mouse anti-α-tubulin (Sigma, T6074).

### 2.13 EdU assay

To evaluate cell proliferation, the BeyoClick™ EdU Cell Proliferation Kit (Beyotime) was employed. The cells were cultured for 24 hours, followed by incubation with EdU labeling (10 μm) medium at 37°C. After fixation, permeabilization, and the Click reaction, nuclei were stained with DAPI. Fluorescence microscopy was employed for image capture, and the proportion of EdU-positive cell nuclei was quantified via ImageJ software to further calculate the cell proliferation rate.

### 2.14 Nile Red staining

Intracellular lipid content was assessed using a lipid fluorescent staining kit (Nile Red Method) (Solarbio, China) in strict accordance with the manufacturer’s protocol. Briefly, the culture medium was aspirated from the cells, which were then rinsed twice with PBS. Subsequently, the cells were fixed with a fixation solution for 10 minutes, followed by an additional PBS rinse. The fixed cells were incubated with the staining solution for 15 minutes under light-protected conditions. After 2–3 rounds of PBS washing, the cells were visualized and imaged using a fluorescence microscope. The fluorescence intensity of the stained cells was further quantified and analyzed using Image J software.

### 2.15 Statistical analysis

Data analysis was performed using R version 4.3.1 and GraphPad Prism 10 software. For intergroup comparative analyses, either unpaired or paired Student’s t-tests were applied, with the selection based on the intrinsic data structure. Correlation assessments were executed through Spearman’s rank correlation coefficient (for non-parametric data) or Pearson’s correlation coefficient (for parametric data). Survival analyses were implemented via Kaplan-Meier Kaplan-Meier curves with log-rank tests and multivariate Cox regression. All experiments were performed in triplicate, with quantitative data presented as mean ± standard deviation (SD). The threshold for statistical significance was defined as two-tailed P < 0.05.

## 3 Results

### 3.1 Pan-cancer expression profiles of *ACACA*

**Figure 1:**
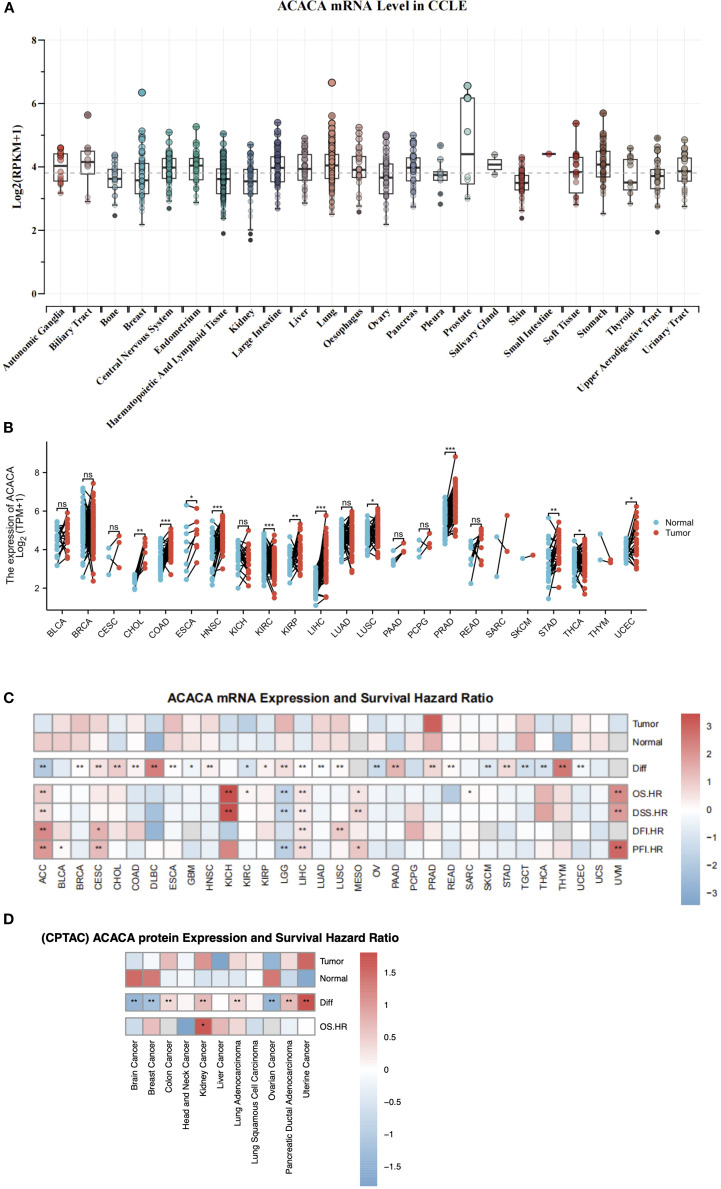
Integrative analysis of *ACACA* expression and survival outcome. **(A)** *ACACA* mRNA expression in different cancer cells form CCLE database. **(B)** Boxplot showing *ACACA* mRNA expression levels in paired tumor samples (red) compared to adjacent normal tissues (blue) in TCGA. **(C)** *ACACA* mRNA expression levels and survival outcome correlation in pan-cancer analysis. A heatmap illustrating that *ACACA* exhibits high expression in the majority of cancers within TCGA and GTEx database. Heatmap integrates hazard ratios (HRs) for OS, DSS, DFI, and PFI. Cox proportional hazards models were used to calculate HRs, with adjustments for age, gender, and stage. Red boxes indicate HR >1 (poor prognosis), blue boxes indicate HR <1 (favorable prognosis). **(D)** ACACA protein expression and overall survival correlation in CPTAC datasets. Significance levels: *p<0.05, **p<0.01, ***p<0.001 (log-rank test with FDR correction).

We first conducted a systematic analysis using the CCLE database, and found significant upregulation of *ACACA* in prostate, lung, stomach, pancreas and liver cell lines, which suggested possible aberrant lipid biosynthesis in these tumors (**Figure 1A**). Subsequent validation in TCGA data also demonstrated significant overexpressions across multiple tumors compared with matched normal controls (**Figure 1B**). Integrated RNA sequencing analysis of TCGA cancer specimens and Genotype-Tissue Expression (GTEx) normal controls confirmed consistent upregulation of *ACACA* in diverse tumors (**Figure 1C**). Specifically, elevated *ACACA* expression pattern was observed in hepatocellular carcinoma (LIHC), stomach adenocarcinoma (STAD), pancreatic ductal adenocarcinoma (PDAC), prostate adenocarcinoma (PAAD), head and neck squamous cell carcinoma (HNSC), non-small cell lung cancer (NSCLC), esophageal carcinoma (ESCA), and cholangiocarcinoma (CHOL). Utilizing transcriptomic and proteomic data from the TCGA and THPA datasets coupled with detailed clinical features, we systematically profiled *ACACA* expressions across diverse TNM stages (**Supplementary Figures 1A–C**). In LUAD, *ACACA* mRNA expression was significantly elevated in the M1-stage tumors, indicating its potential involvement in tumor metastasis.

The expression and catalytic activity of *ACACA* exert direct regulatory control over cellular lipogenesis, which is modulated through multilevel regulation involving transcriptional, post-translational, and metabolic feedback mechanisms. Leveraging proteogenomic data from CPTAC, we identified significant oncogenic dysregulation of ACC1 protein expression across diverse malignancies. Specifically, compared with the corresponding normal controls, quantitative proteomic profiling revealed a substantial increase in protein levels in lung adenocarcinoma, uterine cancer, kidney cancer, colon cancer, and pancreatic ductal adenocarcinoma patients. Conversely, significant downregulation was observed in brain cancer, breast cancer, and ovarian cancer (**Figure 1D**).

### 3.2 Clinical correlation analysis of *ACACA*

**Figure 2:**
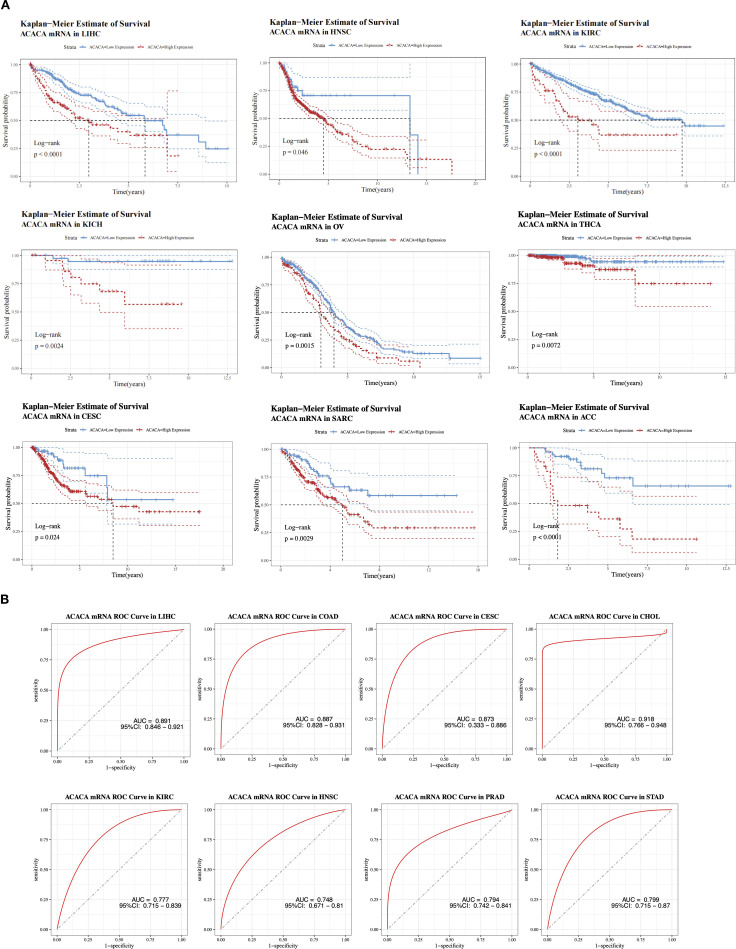
Prognostic value of *ACACA* mRNA expression across cancers. **(A)** Kaplan-Meier survival curves of *ACACA* mRNA expression in multiple TCGA cancer cohorts (LIHC, HNSC, KIRC, KICH, OV, THCA, CESC, SARC and ACC). **(B)** Receiver operating characteristic (ROC) curves demonstrating the diagnostic performance of *ACACA* mRNA expression in distinguishing tumor tissues from normal tissues.

To explore the role of *ACACA* expression in prognostic prediction, Cox regression analyses were carried out to assess its association with different survival endpoints. Integrated analysis revealed that *ACACA* transcript levels were significantly correlated with adverse prognostic indices, with elevated hazard ratios (HRs) for OS, DSS, DFI, and PFI in several tumor types, including LIHC, adrenocortical carcinoma (ACC), mesothelioma (MESO), and uveal melanoma (UVM) (**Figure 1C**). Subsequently, Kaplan-Meier (KM) survival analyses were performed utilizing prognostic data from the TCGA database to visualize survival trends. KM analyses revealed that elevated *ACACA* expression was significantly associated with poorer OS in patients with LIHC, HNSC, ACC, ovarian serous cystadenocarcinoma (OV), sarcoma (SARC), kidney renal clear cell carcinoma (KIRC), cervical squamous cell carcinoma (CESC), kidney chromophobe (KICH) and thyroid carcinoma (THCA) (**Figure 2A**).

Interestingly, in both colon adenocarcinoma (COAD) and lower-grade glioma (LGG) patients (**Supplementary Figures 2A,B**), patients with high *ACACA* expression demonstrated better OS, suggesting a potential context-dependent role for *ACACA* in different cancer types. K-M curves derived from CPTAC clinical proteomic datasets demonstrated significantly worse overall survival in patients with elevated ACC1 protein levels, particularly in those with kidney cancer, liver cancer, uterine cancer, and lung adenocarcinoma (**Supplementary Figures 2C–F**).

Integrative analysis of TCGA transcriptomic data revealed the diagnostic potential of ACACA across malignancies, which was validated through receiver operating characteristic (ROC) curve analysis (**Figure 2B**). *ACACA* demonstrated superior discriminatory capacity in LIHC (AUC = 0.891, 95%CI: 0.846-0.921), COAD (AUC = 0.887, 95%CI: 0.828-0.931), CESC (AUC = 0.873, 95%CI: 0.833-0.886) and CHOL (AUC = 0.918, 95%CI: 0.766-0.948). Additionally, significant tumor discrimination potential was observed in HNSC (AUC = 0.748, 95% CI: 0.671-0.810), KIRC (AUC = 0.777, 95% CI: 0.715-0.839), PRAD (AUC = 0.794, 95% CI: 0.742-0.841), and STAD (AUC = 0.799, 95% CI: 0.715-0.870).

### 3.3 Relationship between *ACACA* gene expression and tumor immune cell infiltration

**Figure 3:**
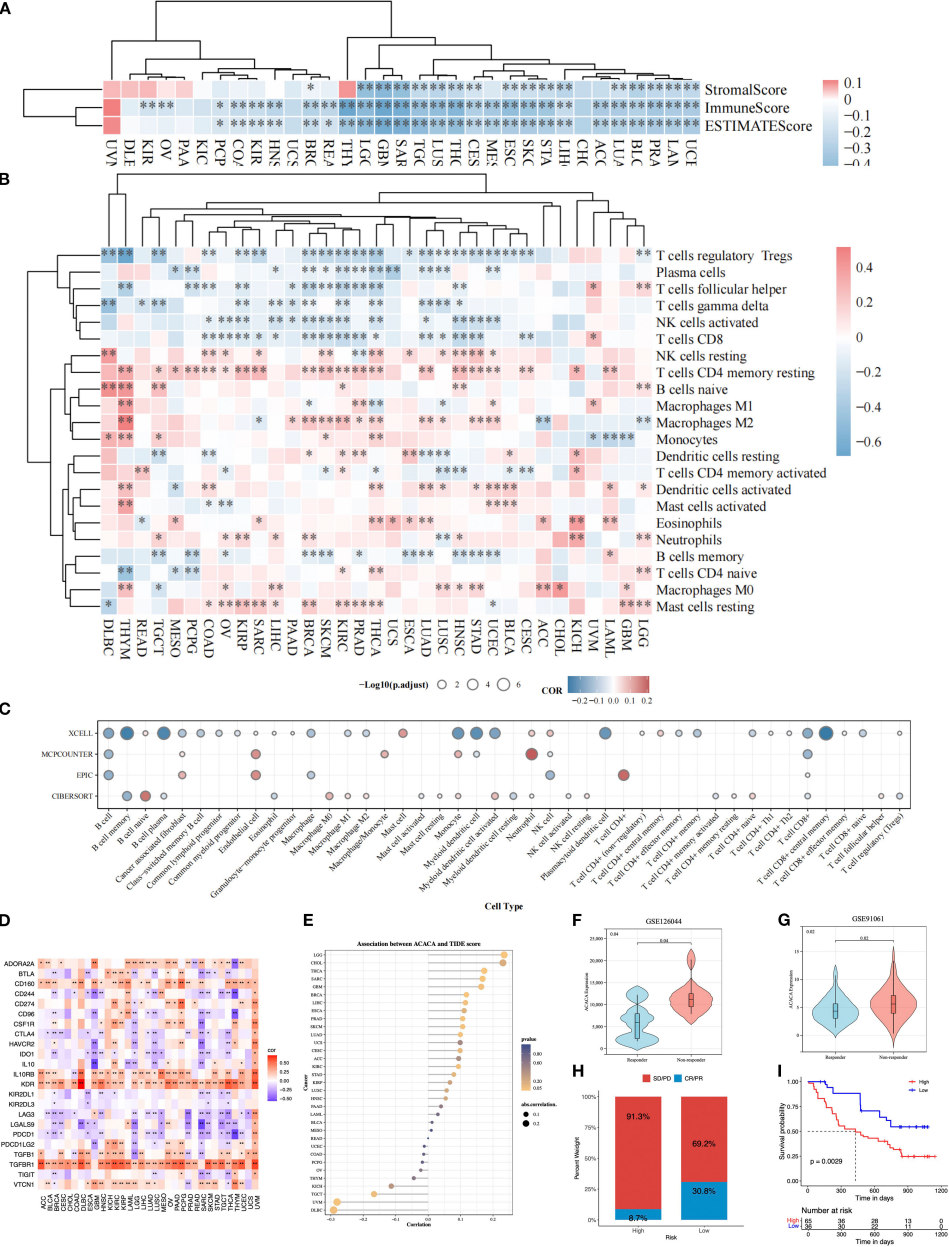
Pan-cancer immune landscape correlated with *ACACA* mRNA expression. **(A)** Correlation of *ACACA* with ESTIMATE score, immune score, and stromal score. **(B)** Immune cell infiltration in tumors stratified by *ACACA* expression (TIMER algorithm). Heatmap colors represent Spearman’s ρ values (red: positive correlation; blue: negative). Significance levels: *p<0.05, **p<0.01, ***p<0.001 **(C)** Circular plot integrating immune cell infiltration results from xCell, MCP-counter, EPIC, and CIBERSORT algorithms. Circle size indicates statistical significance (−log10(p.adjust)); color scale reflects correlation direction (red: positive; blue: negative). **(D)** Association of *ACACA* expression with immune-related genes. **(E)** Correlation between *ACACA* expression and TIDE scores across various cancer types. **(F, G)** Violin plots comparing *ACACA* expression in responders (R) vs. non-responders (NR) from immunotherapy cohorts (GSE126044, GSE91061). **(H, I)** The immune response proportion and survival status of patients in high and low *ACACA* expression groups in GSE91061 cohort.

The tumor microenvironment, composed of stromal elements and immune cells, occupies a central position in governing tumor progression and facilitating immune evasion. Pan-cancer analysis across 33 distinct tumor types, employing ESTIMATE algorithm-derived metrics, revealed a consistently negative association between *ACACA* expression and TME characteristics. Specifically, ACACA expression was significantly negatively correlated with stromal, immune and ESTIMATE scores (**Figure 3A**), suggesting that elevated *ACACA* expression may facilitate the development of an immunosuppressive TME.

Additionally, comprehensive multi-algorithmic analysis (TIMER 2.0, xCell, MCPCOUNTER, EPIC, CIBERSORT) revealed a dual role of *ACACA* in shaping the tumor immune landscape across malignancies. Using the TIMER 2.0 database, we carried out a Spearman correlation analysis to link the *ACACA* transcriptional profiles to the immune cell infiltration data (**Figure 3B**). Systematic interrogation of tumor immune landscapes across 33 malignancies revealed that *ACACA* expression showed significant inverse associations with the infiltration of CD8^+^ cytotoxic T cells and activated NK cells, whereas it positively correlated with resting memory CD4^+^ T cells and M2 macrophage abundance. These associations are particularly apparent in breast cancer, renal cancer, lung adenocarcinoma, and prostate cancer. Further analysis using the xCell algorithm confirmed a significant negative relationship between *ACACA* expression levels and the infiltration of CD8^+^ T cells (**Figure 3C**). EPIC and MCPCOUNTER analyses displayed a significant positive correlation between elevated *ACACA* mRNA expression and increased numbers of endothelial cells, neutrophils, and CD4^+^ T cells (**Figure 3C**). These results indicated that *ACACA* may modulate the populations of diverse immune and stromal cells within the tumor microenvironment, possibly fostering conditions that support tumor development.

Immune checkpoints are pivotal in governing tumor immune evasion, and therapies targeting these axes have markedly transformed cancer therapy. In order to elucidate the interplay between *ACACA*-driven lipid metabolism and immune checkpoint regulation, we performed correlation analyses across 33 cancer types. Strikingly, *ACACA* expression exhibited significant negative correlations with the immune checkpoint-related genes (PDCD1, LAG3, LAGLS9, IDO1, CD244, CTLA4 and TIGIT), while showing positive associations with other genes (TGFBR1, KDR, IL10RB, CD160 and CD274) across most cancer types (**Figure 3D**). Furthermore, the results of TIDE algorithm demonstrated that increased ACACA expression was related to higher TIDE scores in LGG, THCA, SARC, cholangiocarcinoma (CHOL), glioblastoma (GBM) and breast invasive carcinoma (BRCA), which suggested impaired cytotoxic T-cell infiltration and increased immune evasion (**Figure 3E**). In contrast, inverse correlations were observed for UVM, testicular germ cell tumor (TGCT) and diffuse large B-cell lymphoma (DLBC), suggesting tumor-specific regulatory mechanisms. Validation in independent immunotherapy cohorts (GSE126044 and GSE91061) further demonstrated that non-responders (SD/PD) exhibited significantly higher *ACACA* levels than responders (PR/CR) (**Figures 3F, G**). KM survival analysis of the GSE91061 cohort indicated that the patients with high *ACACA* expression patients had an unfavorable prognosis (**Figures 3H, I**). These findings identify *ACACA* as a metabolic orchestrator of immune checkpoint networks and propose its utility as a predictive biomarker for immunotherapy resistance, thereby providing new mechanistic insights into the metabolic-immune interplay in cancer.

### 3.4 Gene set enrichment analysis of *ACACA*

**Figure 4:**
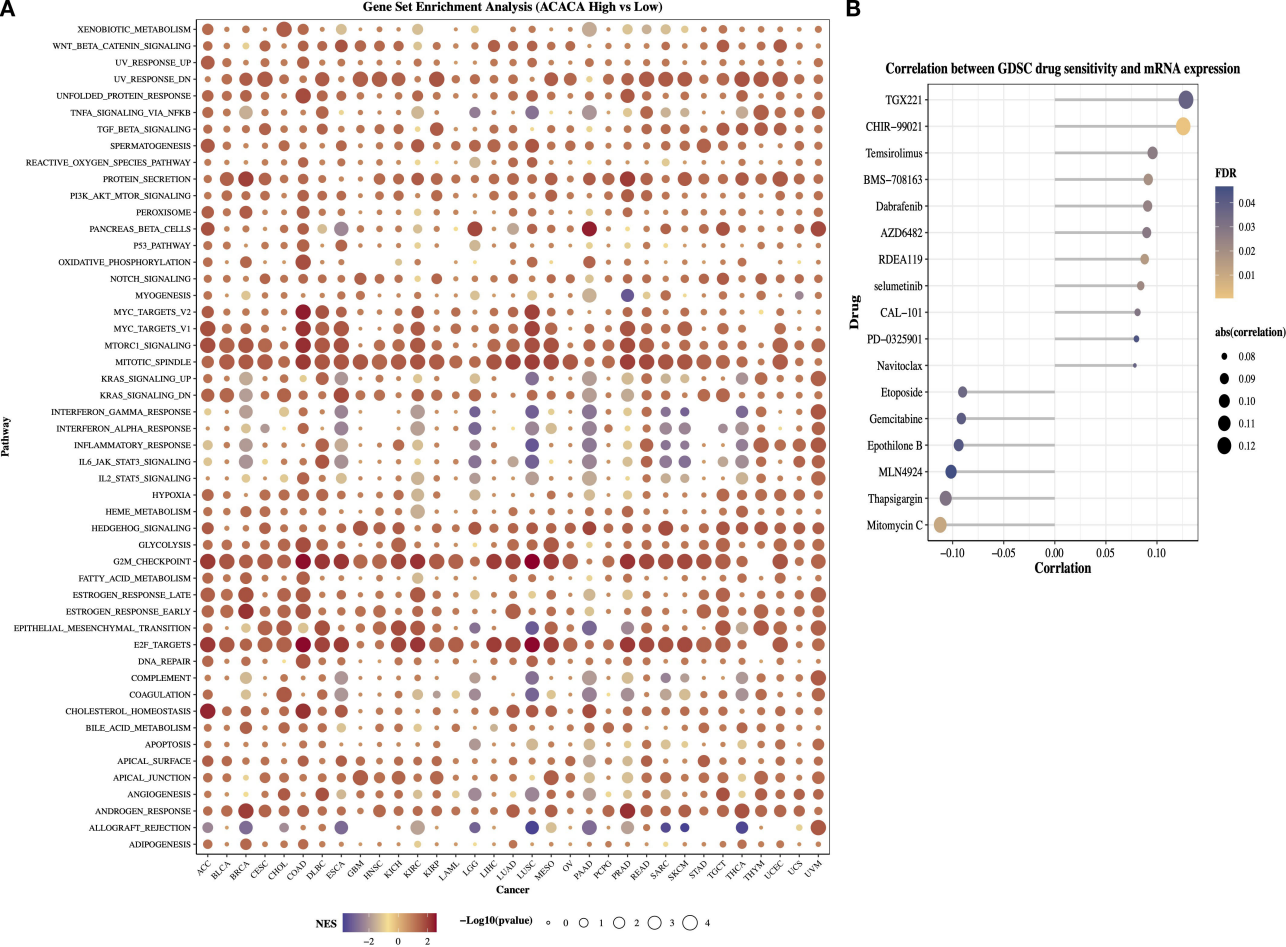
Functional annotation and therapeutic implications of *ACACA* in pan-cancer. **(A)** Gene Set Enrichment Analysis (GSEA) of *ACACA*-high vs. *ACACA*-low tumors (TCGA pan-cancer cohort). **(B)** Correlation between *ACACA* expression and GDSC drug sensitivity in pan-cancer.

To delineate the oncogenic networks modulated by *ACACA*, we performed GSEA on transcriptomic datasets spanning 33 TCGA cancer types, by stratifying patients into high (top 25%) and low (bottom 25%) *ACACA* expression cohorts. Hallmark pathway analysis revealed striking differential enrichment patterns, the high *ACACA* group exhibited significant activation of cell cycle regulatory pathways, including mitotic spindle formation, the G2/M checkpoint, E2F transcriptional targets, and MYC-driven signaling pathway (**Figure 4A**). These findings suggest that *ACACA* may enhance tumor cell proliferation by promoting cell cycle progression and DNA replication. Intriguingly, high *ACACA* expression was inversely correlated with key immunomodulatory pathways, as evidenced by the suppression of interferon-α responses, the inflammatory signaling cascades, and complement activation, particularly in LGG, SARC, PAAD, and lung squamous cell carcinoma (LUSC). These findings imply that *ACACA* might facilitate tumor immune evasion by attenuating critical antitumor immune responses.

The GDSC database provides data on the sensitivity of a broad range of antineoplastic drugs across various cancer cell lines and is widely used for drug-gene association analysis. We next examined the associations between *ACACA* expression levels and dug the IC_50_ values (**Figure 4B**). The efficacy of drugs such as TGX221, CHIR-99021, temsirolimus, and dabrafenib was decreased in patients with higher *ACACA* expression. In contrast, drugs (e.g., mitomycin C, thapsigargin, MLN4924, and epothilone B) showed a negative correlation with *ACACA* expression, suggesting that in pancancer analysis, increased *ACACA* gene expression may contribute to drug sensitivity.

### 3.5 Investigating the function of *ACACA* in lung cancer

**Figure 5:**
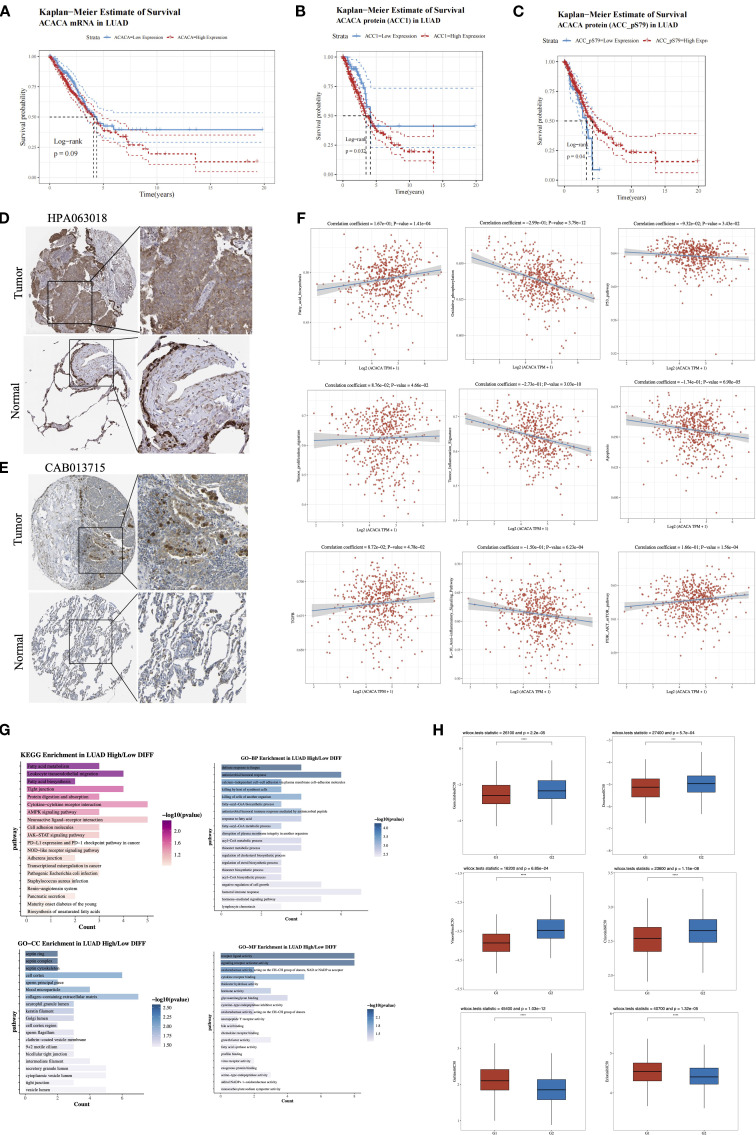
Comprehensive analysis of *ACACA* in lung adenocarcinoma (LUAD). **(A)** Kaplan-Meier survival plots showed the association between *ACACA* mRNA expression and OS in LUAD. **(B)** Kaplan–Meier survival curves reveal the correlation between *ACACA* protein expression levels and OS. **(C)** Kaplan–Meier survival curves reveal the relationship between *ACACA*-S79 protein expression levels and OS. **(D, E)** Representative images showcasing immunohistochemical (IHC) staining of the LUAD samples stained with HPA 063018 and CAB013715 antibody from HPA dataset. **(F)** Correlation between the cell pathway score and *ACACA* expression (assessed using ssGSEA). **(G)** Functional enrichment of *ACACA*-correlated genes in LUAD (KEGG pathway and GO functional annotation). **(H)** Drug sensitivity analysis between high (G1) and low (G2) *ACACA* expression groups.

We subsequently conducted an in-depth analysis of the mRNA and protein expression profiles retrieved from TCGA lung cancer database. Our findings demonstrated that lung cancer patients with elevated *ACACA* mRNA expression had a worse prognosis (**Figure 5A**). Concordantly, KM survival analysis of proteomic data from the TCPA database revealed a significant correlation between high ACC1 protein levels and poorer OS (**Figure 5B**). Interestingly, analysis of ACC1-S79 phosphorylation status suggested that patients with elevated ACC1-S79 expression levels experienced an better prognosis (**Figure 5C**), which is congruent with previous reports indicating that phosphorylation of ACC1 at serine 79 inhibits its enzymatic activity (18, 19). Immunohistochemical analysis via the Human Protein Atlas (HPA) database confirmed significant ACC1 protein upregulation in LUAD tumor tissues (**Figures 5D, E**).

Single-sample gene set enrichment analysis (ssGSEA) of TCGA-LUAD datasets validated significant associations between *ACACA* expression and pathway activity scores (**Figure 5F**). Specifically, *ACACA* upregulation was positively linked to the activation of pathways related to fatty acid synthesis, tumor proliferation, TGF-β signaling, and the PI3K-AKT-mTOR pathway. Conversely, it was negatively correlated with oxidative phosphorylation, apoptosis, and immune-related pathways like the p53 pathway, the tumor inflammatory signature, and the IL-10 anti-inflammatory signaling pathway. The results indicate that *ACACA* may drive proliferation and survival of tumor cells in lung adenocarcinoma by modulating multiple oncogenic and metabolic pathways while inhibiting apoptosis and immune responses. KEGG and GO analyses were conducted to examine biological pathways linked to *ACACA*-related DEGs (**Figure 5G**), the results revealed that *ACACA* expression had associations with key pathways, including fatty acid metabolism, intercellular tight junctions, protein digestion, cytokine-receptor interactions, and the AMPK signaling pathway. Collectively, these findings imply that *ACACA* potentially has a multifaceted role in orchestrating energy balance, modulating immune responses, and driving cancer progression.

Through a systematic evaluation of the relationship between common driver gene mutations in lung cancer and the expression level of *ACACA*, we observed frequent upregulation of *ACACA* expression in patients harboring TP53 mutations or EGFR mutations (**Supplementary Figure 3**). To further elucidate the therapeutic implications of *ACACA* expression, we analyzed drug sensitivity using IC_50_ value as a quantitative pharmacodynamic parameters. Our analysis demonstrated that the high *ACACA* expression group exhibited markedly reduced IC_50_ values (p < 0.05) for both chemotherapeutic agents (docetaxel, gemcitabine, vinorelbine) and the ALK-inhibitor crizotinib when compared with the low expression group, indicating enhanced therapeutic sensitivity to these treatments (**Figure 5H**). Interestingly, by integrating mRNA expression data of EGFR-mutated lung cancer patients from TCGA and the drug sensitivity data in the GDSC database, we found that for small molecule EGFR-TKI drugs (e.g., gefitinib, erlotinib), high *ACACA* expression in the EGFR-mutated group was associated with increased IC_50_ value. These findings suggest that *ACACA*-mediated metabolic reprogramming may contribute to the development of resistance to EGFR-TKI therapy in LUAD.

### 3.6 scRNA-seq analysis reveals the role of *ACACA* in LUAD TME

**Figure 6:**
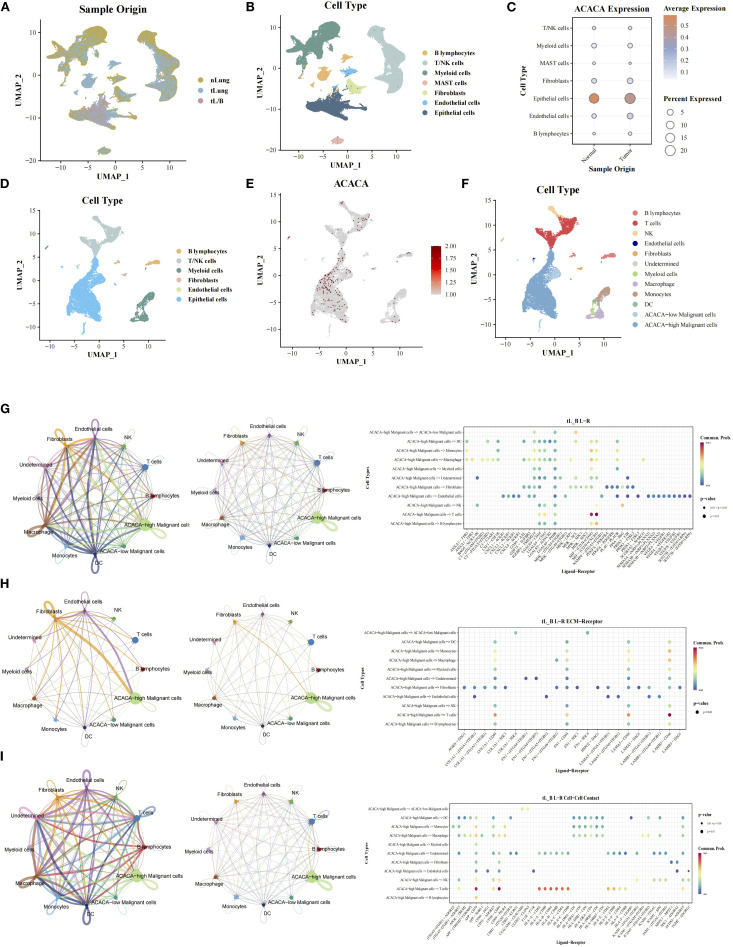
Single-cell RNA sequencing analysis of *ACACA* in LUAD (GEO: GSE131907). **(A)** UMAP projection of all cells from different samples. **(B)** Cell cluster annotation: UMAP visualization of 7 major cell types (epithelial cells, fibroblasts, T/NKcells, MAST cells, B cells, Myeloid cells, endothelial cells). **(C)** The expression of *ACACA* in single cell from tumor and normal tissues. **(D)** Cell cluster annotation of advanced-stage samples: UMAP visualization of 6 major cell types (epithelial cells, fibroblasts, T/NKcells, B cells, Myeloid cells, endothelial cells). **(E)** Expression of *ACACA* across the single-cell landscape. UMAP color scale reflects normalized expression levels (log2(CPM + 1)), with red indicating high expression. **(F)** UMAP visualization of tumor cells segregated into *ACACA*-high (red) and *ACACA*-low (blue) groups. **(G-I)** Cellchat analysis of interaction between cell subsets via **(G)** Secreted Signaling, **(H)** ECM-Receptor and **(I)** Cell-Cell Contact.

To characterize the role of *ACACA* in the LUAD TME, we integrated single-cell transcriptomic datasets from normal lung (nLung), early-stage (tLung), and advanced tumor (tL/B) tissues derived from the GSE131907 dataset (20) (**Figure 6A**). Following rigorous quality control and batch correction, unsupervised clustering analysis identified seven major cell types: B cells, T/NK cells, myeloid cells, mast cells, fibroblasts, endothelial cells, and epithelial cells (**Figure 6B**), with annotation based on known marker genes from previous article (**Supplementary Figure 4D**). *ACACA* expression was predominantly localized to epithelial cells, with comparable expression levels across tumor and normal tissues (**Figure 6C**). In advanced tumors (tL/B), subclustering analysis of the epithelial cell compartment was carried out, which further stratified the epithelial cells into *ACACA*-high and *ACACA*-low subsets (**Figures 6D–F**). Subsequent CellChat analysis revealed enhanced interactions between *ACACA*-high tumor cells and CD8^+^ T cells, primarily via secreted signaling pathways like the macrophage migration inhibitory factor (MIF) axis (MIF-CD74+CXCR4, MIF-(CD274+CD44)) (**Figure 6G**). The LAMB3-DAG1 pathway is dominant in ECM-receptor interactions (**Figure 6H**), and the APP (APP-CD74) and CD99 (CD99-CD99) pathways were the major contributors to cell-cell contact pathways. These findings collectively suggest that *ACACA* may orchestrate fatty acid metabolic reprogramming to potentiate tumor-immune crosstalk through specific signaling networks, thereby fostering the establishment of an immunosuppressive niche and metastatic progression (**Supplementary Figure 4**).

### 3.7 *ACACA* enhances tumor self-renewal and drug resistance in lung cancer cells

**Figure 7:**
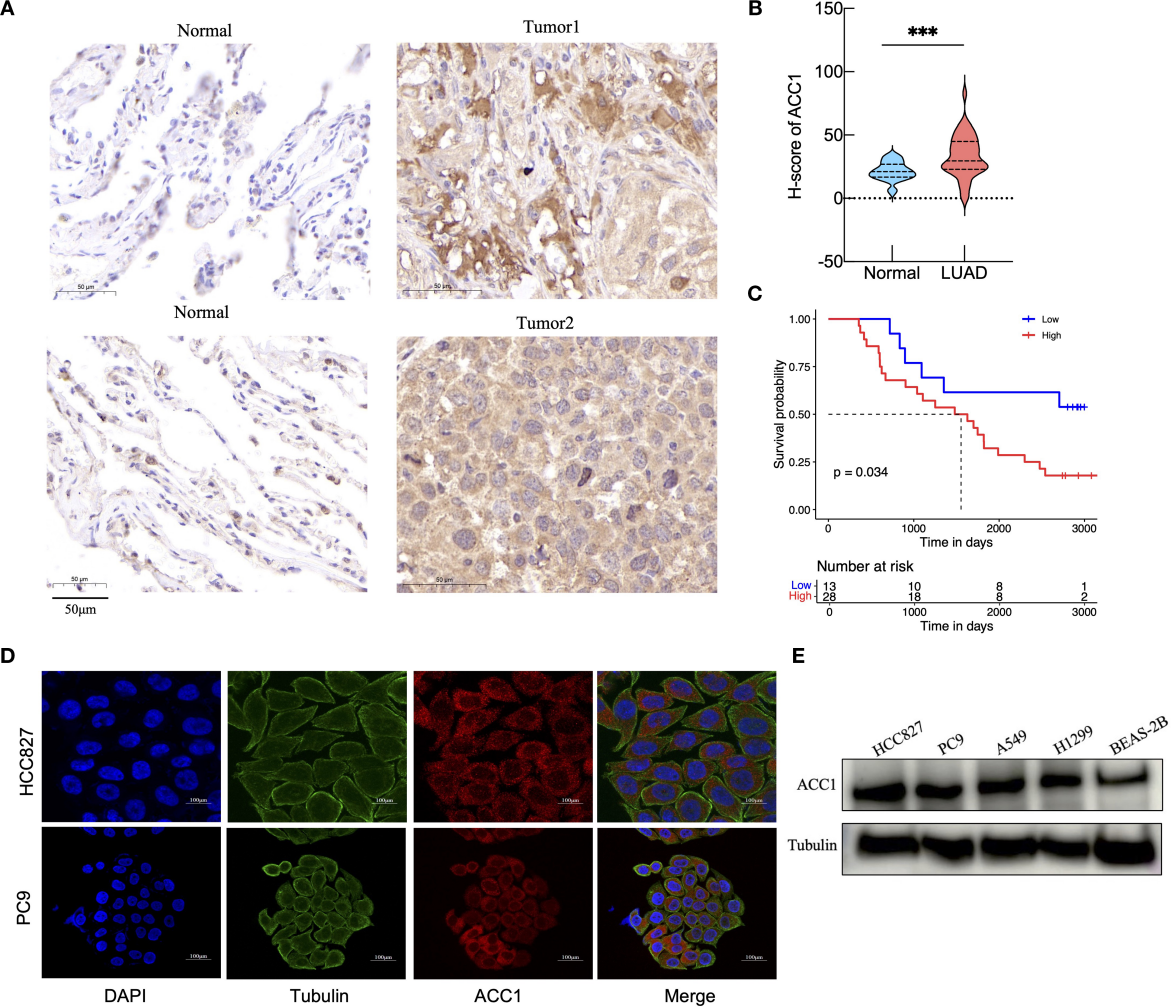
Validation of ACACA expression levels in clinical tissue samples and cell lines. **(A)** Representative images of ACC1 staining in LUAD tumor tissue and paracancerous tissue from clinical samples. Scale bar: 50 mm. **(B)** Analysis of ACC1 expression in tumor tissues and normal tissues. **(C)** The Kaplan-Meier’s survival curve according to ACC1 expression levels. **(D)** Subcellular localization and expression intensity of ACC1 in HCC827 and PC9 cells. **(E)** The relative expression of ACC1 in LUAD cell lines (HCC827, PC9, A549, and H1299) examined by western blot; human bronchial epithelial (BEAS-2B) cell as control. The symbols indicate the level of significance: *** for p < 0.001.

Immunohistochemical analysis of ACC1 protein expression was performed on paraffin-embedded sections from 41 LUAD patients (**Figure 7A**). The expression of ACC1 in tumor tissues was significantly elevated compared to the adjacent normal lung tissues (**Figure 7B**). Using the median H-score as a cutoff, patients were divided into high (n =28) and low (n=13) ACACA expression groups. Kaplan Meier (K-M) survival analysis indicated that high levels of ACC1 suffered poorer OS (**Figure 7C**). And then, immunofluorescence staining of LUAD cell lines (HCC827 and PC9) revealed that ACC1 protein is predominantly localized within the cytoplasm (**Figure 7D**), which is consistent with its central role in fatty acid metabolism. Bioinformatic analyses revealed the significant influence of *ACACA* on cell proliferation across a variety of malignant tumors. For a deeper investigation of its biological function in LUAD, ACC1 protein levels were quantified in lung cancer cell lines, revealing significantly higher protein expression in most LUAD cell lines relative to normal bronchial epithelial cells (**Figure 7E**).

**Figure 8:**
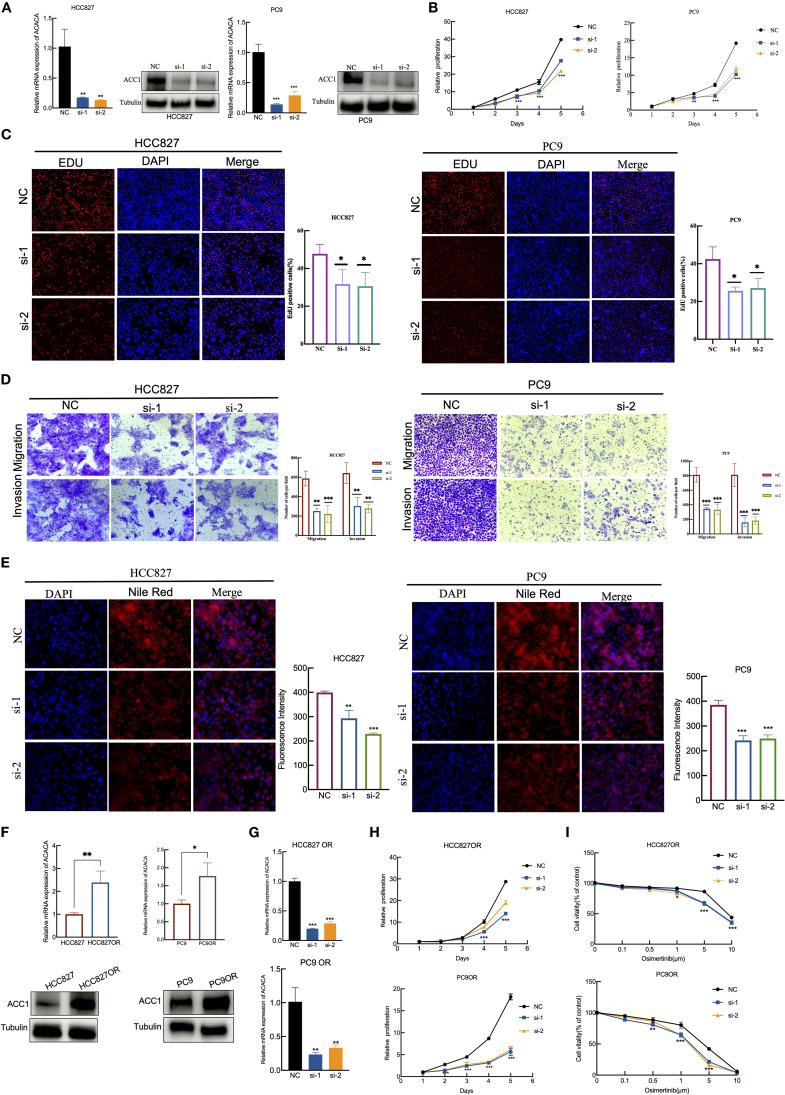
ACACA drives proliferation, migration, and EGFR-TKI resistance in lung cancer cells. **(A)** RT-qPCR and WB verification of the silent efficiency of *ACACA* in HCC827 and PC9 cells. **(B)** Proliferation of HCC827 and PC9 cells transfected with two siRNAs targeting *ACACA* and scrambled control was determined using CCK8 assays. **(C)** EdU assay was performed to assess the proliferative capacity in HCC827 and PC9 cells treated with NC or *ACACA* siRNA. Red: EdU+ proliferating cells; blue: DAPI. Representative images and quantificative analysis of the transwell assay using HCC827 and PC9 transfected *ACACA* siRNA. **(D)** Representative images and quantificative analysis of the transwell assay using HCC827 and PC9 transfected *ACACA* siRNA. **(E)** Nile red staining was used to detect the effect of *ACACA* knockdown on intracellular lipid accumulation. The mean flurescence intensity were assessed by Image J software. **(F)** *ACACA* expression levels in parental cells (HCC827 and PC9) and Osimertinib-resistant cells (HCC827OR and PC9OR). Measured by RT-qPCR and WB assays. **(G)** RT-qPCR for the expression of ACACA in HCC827OR and PC9OR cells transfected with *ACACA* siRNA. **(H)** CCK8 proliferation assays in HCC827OR and PC9OR cells treated with *ACACA* siRNA and Control siRNA. **(I)** Sensitivity to osimertinib in HCC827OR and PC9OR cells following transfection with control or *ACACA* siRNAs. The symbols indicate the level of significance: * for p < 0.05, ** for p < 0.01, *** for p < 0.001, and **** for p < 0.0001; ns denotes non-significance.

Then we employed RT-qPCR and western blotting to validate the silencing efficiency of siRNAs targeting of *ACACA* (**Figure 8A**). The results of the CCK-8 assays and EdU assays showed that *ACACA* silencing decreased the proliferative ability of HCC827 and PC9 cells (**Figures 8B, C**). Additionally, cells transfected with *ACACA* siRNA notably impaired migration and invasion capacities (**Figure 8D**). Nile red staining further demonstrated that cellular lipids are significantly lowered by *ACACA* silencing, compared with control cells (**Figure 8E**). Osimertinib-resistant cells (HCC827OR and PC9OR) were established by exposing parental cells to gradually escalating doses of osimertinib over an extended period. CCK8 assays showed that the IC_50_ values of osimertinib in HCC827OR and PC9OR were 10.87 μm and 5.509 μm, respectively, both significantly higher than those in sensitive cells (**Supplementary Figure 5E**). Then we found that *ACACA* expression was upregulated in Osimertinib-resistant cells through qPCR and WB (**Figure 8F**). Using *ACACA*-specific siRNA markedly inhibit cell proliferation of HCC827 OR and PC9 OR cells (**Figures 8G, H**), and might increase the sensitivity of drug-resistant cells to Osimertinib (**Figure 8I**). These findings suggest that *ACACA* may be a key factor regulating the biological behavior of tumor cells, and a similar effect of *ACACA* silencing effect were observed in sarcoma cells. According to the CCK8, transwell and Nile Red staining assays, *ACACA* notably increased the proliferation, migration and lipid synthesis capabilities of MG63 and U2OS cells (**Supplementary Figures 5A–D**). Inhibiting *ACACA* effectively suppressed fatty acid synthesis and tumor growth.

## 4 Discussion

*ACACA* acts as a key regulator of fatty acid synthesis and energy homeostasis (21, 22). However, its landscape across different cancers and specific mechanism in tumor immune microenvironment remains a mystery. In this study, we utilized a multi-omic approach to amalgamate publicly accessible expression and survival data from cancer patients, thereby profiling the *ACACA* expression landscape. We found that *ACACA* exhibits significant prognostic value in multiple cancers. Specifically, high *ACACA* mRNA expression correlated with poorer outcomes in LIHC, KIRH, OV, and SARC, whereas it predicted improved prognosis in COAD and LGG. Moreover, clinical specimens from 41 LUAD patients confirmed that high ACACA expression adversely impacts long-term survival. We further demonstrated that *ACACA* was closely related with tumor immune microenvironment and could serve as a immunotherapeutic biomarker. Single-cell analysis in lung cancer highlighted its role in activating oncogenic signaling pathways. Subsequent functional experiments showed that targeted knockdown of *ACACA* can reduce the proliferation, metastasis, and lipid synthesis capabilities of tumor cells. Obviously, these results suggest that *ACACA* could serve as potential prognostic and immunotherapeutic biomarker across cancers especially lung adenocarcinoma.

As a key metabolic regulatory enzyme, *ACACA*’s oncogenic activity is affected by the metabolic heterogeneity of tumor cell subpopulations, and depends on hypoxia and nutrient conditions in the microenvironment. Furthermore, the process by which ACC1 catalyzes the conversion of acetyl-CoA to malonyl-CoA may deplete the acetyl group pool available for protein acetylation, thereby affecting cell proliferation, growth, and migration. In hepatocellular carcinoma, cells exhibit abnormally active lipid synthesis capacity, and the expression of *ACACA* is positively correlated with tumor malignancy (18, 23). In cholangiocarcinoma, inhibiting *ACACA* enhances the acetylation of HSP90 so as to hinder the proliferation and migration of tumor cells (24). However, in breast cancer, inhibition of *ACACA* leads to the enhancement of Smad2 acetylation, ultimately resulting in epithelial-mesenchymal transition (EMT) and metastasis (25). This observation is presumably attributed to tumor-specific metabolic dependencies and inherent heterogeneity in the tumor immune landscape. ACC1 harbors multiple phosphorylation sites (10), a recent study reported that under energy-depleted conditions, AMP-activated protein kinase (AMPK) phosphorylates ACC1 at serine 79, which prevents its polymerization and activation (26). AMPK activators can enhance immunotherapy efficacy by reversing T-cell exhaustion (27). Consequently, modulating ACC1 expression or its phosphorylation status may represent a promising therapeutic strategy to improve cancer outcomes.

Within the tumor microenvironment, tumor and immune cells compete for nutrients essential for proliferation and anabolism (28, 29). Our analysis showed that *ACACA* expression negatively correlates with CD8^+^ T cells and M1 macrophages infiltration, while positively associating with M2 macrophages. This pattern suggests that ACC1-driven metabolic reprogramming fosters an immunosuppressive TME by altering nutrient allocation, thereby impairing immune surveillance (30, 31). Furthermore, *ACACA* expression is negatively correlated with key immune checkpoint genes (PDCD1, TIGIT, and CTLA4), the high *ACACA* expression group had higher TIDE score. Therefore, high *ACACA* levels are linked to reduced responsiveness to immunotherapies such as anti-PD1 and anti-CTLA4. A previous study has promoted an immune evasion mechanism in head and neck squamous cell carcinoma that activated ACC1 reduces H3K27 acetylation, resulting in reduced galectin-9 expression. Galectin-9 binds to immune checkpoint proteins TIM-3 and PD-1, suppressing the production of cytotoxic cytokines by T cells and facilitating T cell apoptosis (32). Therefore, high ACC1 levels are linked to reduced responsiveness to immunotherapies such as anti-PD1 and anti-CTLA4. Consequently, targeting ACC1 or its downstream metabolic pathways could be a promising strategy for reprogramming tumor metabolism, enhancing immune cell functionality, and improving the efficacy of immune checkpoint blockade therapies.

Our functional experiments mainly focused on lung cancer and sarcoma cells. *In vitro* experiments, when *ACACA* was knocked down via siRNAs, lung adenocarcinoma and sarcoma cells exhibited significantly reduced proliferation, invasion, and metastatic ability. Recent research has demonstrated that in lung cancer cells, long non-coding RNA CTD-2245E15.3 (33) or STAT3 (34) promote the transcription of *ACACA*, thereby enhancing the proliferative and metastatic potential of tumor cells. In preclinical models, the pharmacological suppression of ACC1 effectively decreases the proliferation and metastasis of lung adenocarcinoma cells, such as the A549 and H1299 cell lines (35). However, ACC1 does not only influence lung cancer cells through intrinsic cellular mechanisms. Interestingly single-cell analysis of LUAD revealed that *ACACA*-high epithelial cells interact with CD8^+^ T cells via MIF signaling pathway while activates LAMB3-DAG1-mediated ECM remodeling, which makes *ACACA* a conductor of metabolic-immune-stromal crosstalk during metastatic progression. In our study, we also found that *ACACA* expression is related to mutations of common driver genes in NSCLC, which agrees with recent research that common driver gene mutations in lung cancer, such as KRAS, EGFR, ROS1 and ALK, are closely related to metabolism. For example, KRAS-mutant NSCLC relies on *de novo* fatty acid synthesis and phospholipid remodeling to combat oxidative stress and evade ferroptosis (36). In MYC-translocated multiple myeloma, ACC1 exhibits abnormal overexpression. Its selective inhibition disrupts lipid homeostasis, induces endoplasmic reticulum (ER) stress and impairs malignant cell survival (37). The PI3K/AKT/mTOR pathway downstream of EGFR is not only a key signaling axis for EGFR-driven oncogenesis but also a core pathway for regulating lipid synthesis. Also, our study found that *ACACA* mRNA expression is significantly higher in lung cancer patients with EGFR mutations than those with wild-type EGFR. Additionally, *ACACA* expression is significantly upregulated in osimertinib-resistant cells, and targeted knockdown of *ACACA* markedly enhances the sensitivity of these resistant cells to osimertinib. This observation implies that *ACACA*-mediated metabolic reprogramming may be involved in osimertinib resistance of lung adenocarcinoma; however, the current findings alone are insufficient to establish a definitive causal relationship between ACACA and this drug-resistant phenotype.

Targeting ACC1 holds promises as a novel therapeutic approach for tumors with dysregulated metabolism. Current small-molecule inhibitors such as TOFA, ND-630, ND-654 and Soraphen, demonstrate antitumor potential in various solid tumors and hematological malignancies (38). ND630 is an allosteric protein inhibitor of ACC1,that prevents the dimerization of ACC1 and inhibits its enzymatic activity. In prostate cancer, ND630 regulates the expression of circKIF18B_003, thereby achieving the regulation of *ACACA* and lipid reprogramming (39). Additionally, ND630 can alleviate hepatic steatosis and regulate dyslipidemia in obese rats by inhibiting ACC1 (40). ND654 is a potent ACC inhibitor with liver-selective targeting. When cirrhotic rats with liver cancer are administered 10 mg/kg of ND654 via daily intragastric gavage, it can increase the survival rate of the rats and enhance the efficacy of sorafenib (18). Future studies are needed to integrate metabolomics, proteomics, and singe-cell omics technologies to explore biomarkers, identify appropriate indications and on-target metabolic side-effects for *ACACA*-targeted therapies.

## 5 Conclusions

In conclusion, our study elucidates a comprehensive role of *ACACA* across cancers, not only as a canonical lipogenic enzyme but also dynamic regulator of the cancer ecosystem that integrates metabolic flux with immune evasion and stromal remodeling. *ACACA* can serve as a prognostic and immunotherapeutic biomarker. However, this study has several limitations: the number of clinical samples obtained is relatively small, metabolomics-related data are lacking, and an *in vivo* drug-resistant model has not yet been established to support mechanistic investigations. In the future, we plan to include multi-center, multi-cohort case data to improve the generalizability and reliability of the study conclusions; combine technologies such as single-cell sequencing, spatial transcriptomics, untargeted and targeted metabolomics technologies to analyze verify its role in drug-resistant mechanisms and potential therapeutic value.

## Data Availability

The original contributions presented in the study are included in the article/**Supplementary Material**. Further inquiries can be directed to the corresponding authors.
