# Supplementary material for: Integrated single-cell and bulk RNA sequencing analysis reveals ACACA as a potential prognostic and immunotherapeutic biomarker across cancers
Source: Front Immunol. 2025 Oct 15;16:1599223. doi: 10.3389/fimmu.2025.1599223 (PMC12568587; doi:10.3389/fimmu.2025.1599223)
Supplement: Supplementary file 1 [file SupplementaryFile1.docx]

**Supplementary Materials.**

**Supplementary Figure 1**

**
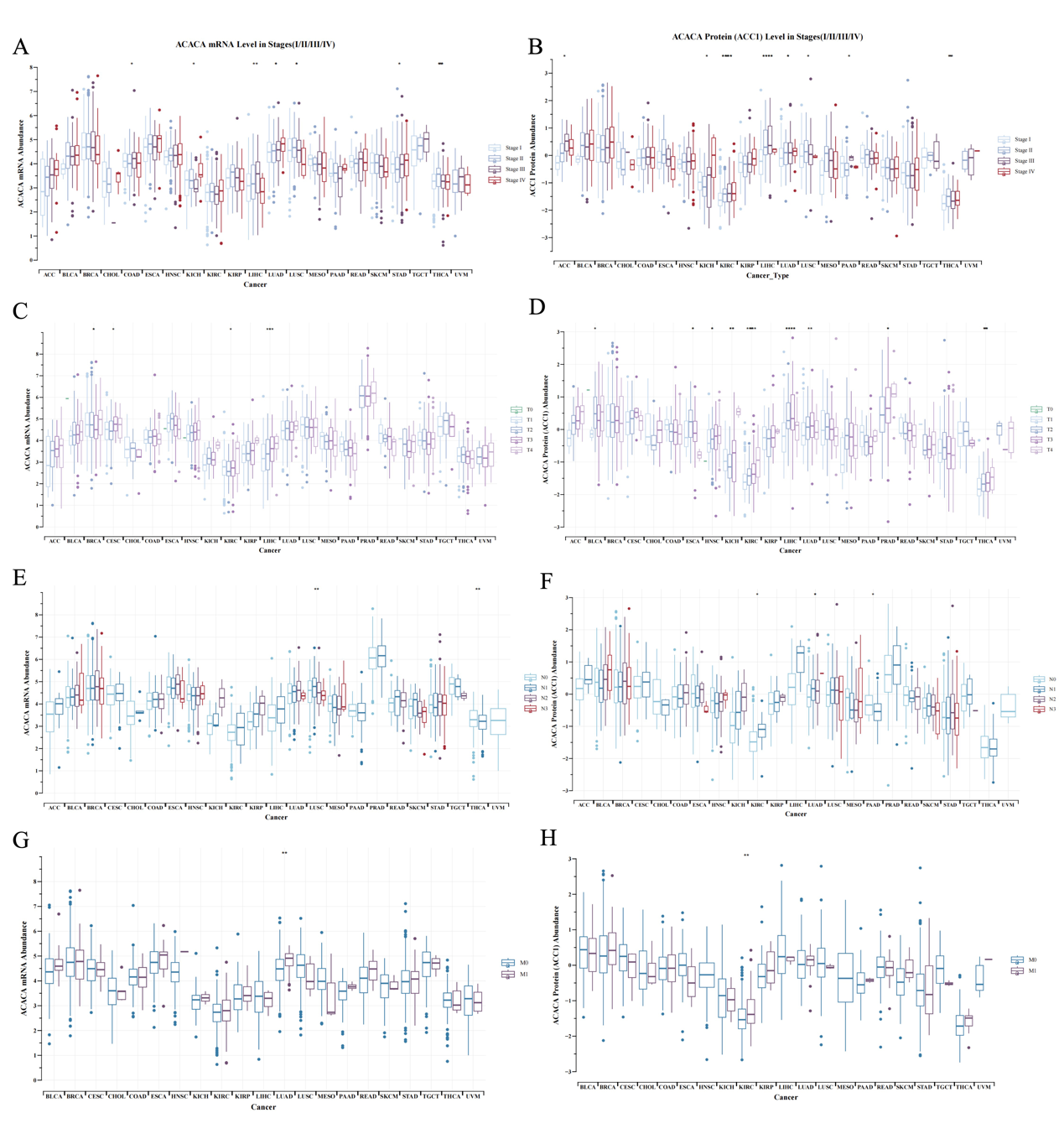
** **Supplementary Figure 1.** Pan-cancer association of ACACA expression with tumor stage and metastasis. (A-B) Associations between ACACA mRNA or protein expressionand tumor stages (Stage I–IV) in TCGA cohorts. (C-D) Associations between ACACA mRNA or protein expression expression and tumor size (T0–T4) in TCGA cohorts. (E-F) Associations between ACACA mRNA or protein expression expression and lymph node involvement (N_0_–N_3_) in TCGA cohorts. (G-H) Associations between ACACA mRNA or protein expression expression and distant metastasis (M_0_ vs. M_1_) in TCGA cohorts.

**Supplementary Figure 2**

**Supplementary Figure2.** Kaplan-Meier survival curves of ACACA mRNA or Protein expression in cancers. A-B. Kaplan-Meier survival curves of ACACA mRNA expression in COAD and LGG (TCGA database). C-F Kaplan-Meier survival curves of ACACA protein expression in Kidney Cancer, Liver Cancer, Uterine Cancer and Lung Adenocarcinoma (CPTAC database).

**Supplementary Figure3**

**Supplementary Figure3** Correlation between ACACA expression and genetic abnormalities of driver genes in LUAD. ACACA expression level in wild type and mutated group of TP-53 (A), EGFR (B), KRAS (C), ALK (D), BRAF (E), ROS (F). (*p<0.05, **p<0.01, ns, not significant)

**Supplementary Figure4**


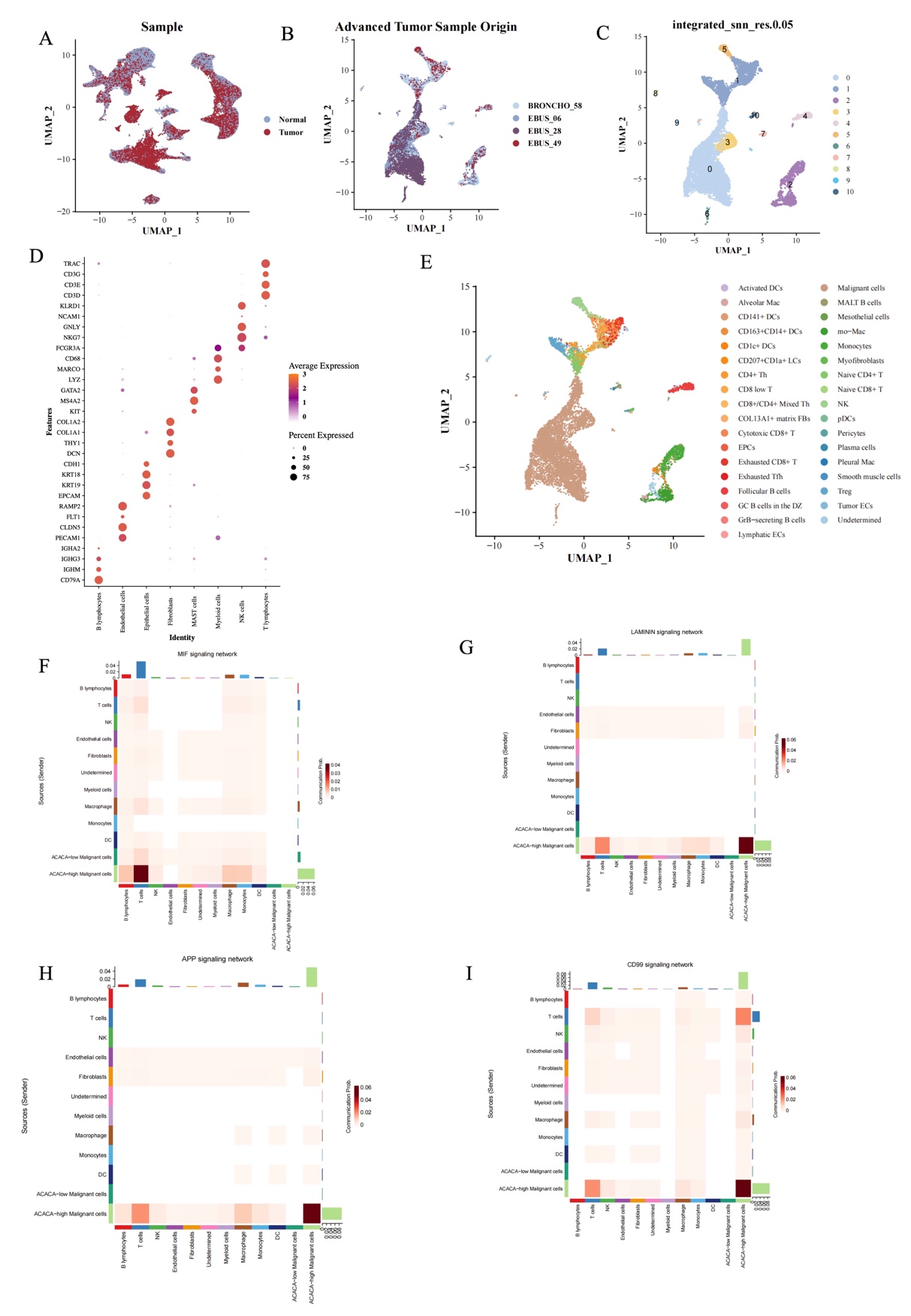


**Supplementary Figure4.** Single-cell RNA sequencing analysis of ACACA in LUAD (GEO: GSE131907). (A) UMAP of cell distribution of tumor cells and normal cells. (B) UMAP of source of advanced-stage tumor samples. (C) UMAP analysis of all cells in advanced-stage tumor samples. (D)Marker gene expression for all annotated cell types. (E) UMAP plot displaying cell type annotation in advanced-stage tumor samples. (F-I) Function roles in cell-cell communication in advanced tumor samples.

**Supplementary Figure5**


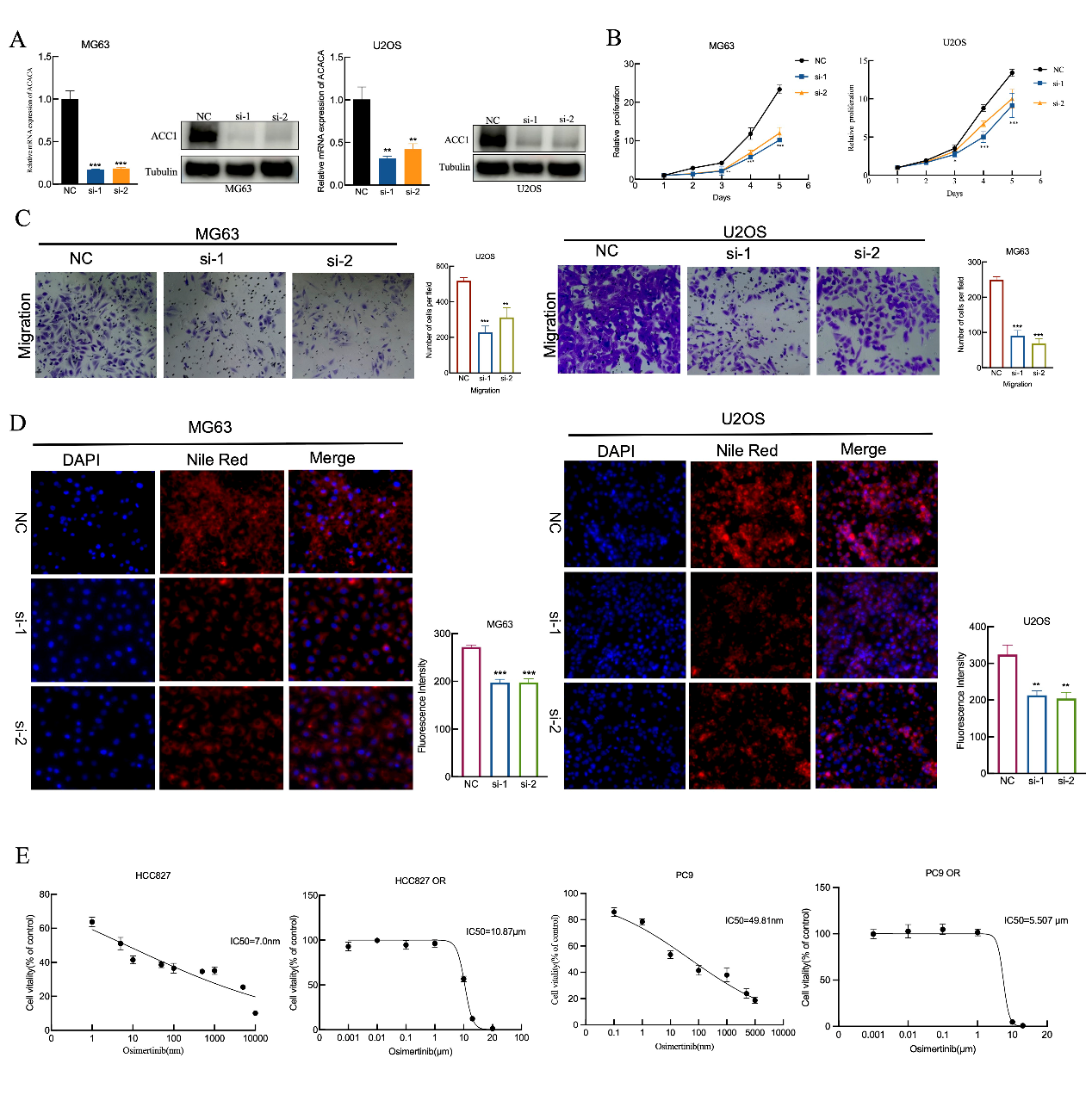


**Supplementary Figure 5.** Validation of the functions of ACACA in sarcoma by in vitro cell experiments. (A) RT-qPCR and WB verification of the silent efficiency of ACACA in MG63 and U2OS cells. (B) Proliferation of MG63 and U2OS cells transfected with two siRNAs targeting ACACA and scrambled control was determined using CCK8 assays. (D) Representative images and quantificative analysis of the transwell migration assay using MG63 and U2OS transfected with ACACA siRNA. (E) Nile red staining was used to detect the effect of ACACA knockdown on intracellular lipid accumulation. The mean flurescence intensity were assessed by Image J software. (F) Proliferation inhibition curves of NSCLC cells after treatment with Osimertinib at indicated concentrations.
